# Glycogen synthesis correlates with androgen-dependent growth arrest in prostate cancer

**DOI:** 10.1186/1471-2490-5-6

**Published:** 2005-03-24

**Authors:** Joachim B Schnier, Kayoko Nishi, Paul H Gumerlock, Frederic A Gorin, E Morton Bradbury

**Affiliations:** 1 Department of Biochemistry and Molecular Medicine, Tupper Hall, University of California, One Shields Avenue, Davis, CA 95616, USA; 2 Cancer and Molecular Research Laboratory, University of California Davis Cancer Center, 4501 X Street, Sacramento, CA 95817, USA; 3 Center for Neuroscience, University of California at Davis, Davis, CA, USA; 4 Los Alamos National Laboratories, Biosciences Division, Los Alamos, NM 87545, USA

## Abstract

**Background:**

Androgen withdrawal in normal prostate or androgen-dependent prostate cancer is associated with the downregulation of several glycolytic enzymes and with reduced glucose uptake. Although glycogen metabolism is known to regulate the intracellular glucose level its involvement in androgen response has not been studied.

**Methods:**

We investigated the effects of androgen on glycogen phosphorylase (GP), glycogen synthase (GS) and on glycogen accumulation in the androgen-receptor (AR) reconstituted PC3 cell line containing either an empty vector (PC3-AR-V) or vector with HPV-E7 (PC3-AR-E7) and the LNCaP cell line.

**Results:**

Androgen addition in PC3 cells expressing the AR mimics androgen ablation in androgen-dependent prostate cells. Incubation of PC3-AR-V or PC3-AR-E7 cells with the androgen R1881 induced G1 cell cycle arrest within 24 hours and resulted in a gradual cell number reduction over 5 days thereafter, which was accompanied by a 2 to 5 fold increase in glycogen content. 24 hours after androgen-treatment the level of Glucose-6-P (G-6-P) had increased threefold and after 48 hours the GS and GP activities increased twofold. Under this condition inhibition of glycogenolysis with the selective GP inhibitor CP-91149 enhanced the increase in glycogen content and further reduced the cell number. The androgen-dependent LNCaP cells that endogenously express AR responded to androgen withdrawal with growth arrest and increased glycogen content. CP-91149 further increased glycogen content and caused a reduction of cell number.

**Conclusion:**

Increased glycogenesis is part of the androgen receptor-mediated cellular response and blockage of glycogenolysis by the GP inhibitor CP-91149 further increased glycogenesis. The combined use of a GP inhibitor with hormone therapy may increase the efficacy of hormone treatment by decreasing the survival of prostate cancer cells and thereby reducing the chance of cancer recurrence.

## Background

Androgen withdrawal leads to apoptosis of normal prostate cells and is the principal therapy to treat advanced prostate cancer [for a review, [1]]. Metabolic events known to be associated with androgen withdrawal are reduction in glucose uptake, downregulation of several glycolytic enzymes and of some key enzymes of the pentose-phosphate shunt [2-5]. Androgen withdrawal led to transcriptional downregulation of the pyruvate dehydrogenase E1 alpha (PDH E1α) gene in rat ventral prostate and in PC3 prostate cancer cells transiently transfected with the androgen receptor. Reduced transcription of PDH E1α is associated with a reduction of the glucose oxidative pathway [6]. In contrast, androgen stimulated CO_2 _production derived from glucose [2]. These results suggest that glucose transporters and several catabolic enzymes are regulated in an androgen-dependent manner.

Glycogen metabolism is regulated by intermediates of glycolysis, by covalent modification and by glycogen and purines. The two major enzymes GS and GP are controlled by phosphorylation and allosterically by effector molecules [7-9]. Glycogen synthase (GS) in its phosphorylated form is inactive but can be activated allosterically by G-6-P. This can facilitate the dephosphorylation by a glycogen-bound PP1-type phosphatase to the active form [10,11]. Active GS is inactivated by phosphorylation by several important protein kinases: casein kinase II, calmodulin-dependent kinases, protein kinase A (PKA), protein kinase C (PKC) [12,13]. Glycogen synthase kinase 3 (GSK-3), a major kinase inactivating GS, phosphorylates several sites on GS but only when GS has been phosphorylated at other sites [14]. Partial dephosphorylation of a specific N- or C-terminal residue increases the sensitivity of GS to activation by G-6-P [15].

Glycogen phosphorylase (GP) also exists in two forms, the active phosphorylated a-form (GP-a) and the inactive b-form (GP-b). cAMP and calcium stimulate the activation of GP through PKA and phosphorylase (PHOS) kinase, which seems to be the only kinase phosphorylating GP [16]. Muscle GP is allosterically activated by the binding of AMP, whereas G-6-P and glucose are allosteric inhibitors [9].

We have recently shown that the cyclin-dependent kinase inhibitor flavopiridol, which is in clinical trials as an anticancer agent, is also a potent GP inhibitor and binds to the purine-nucleotide inhibitor-binding site of GP [17,18]. Inhibition of glycogen degradation by the specific GP inhibitor CP-91149 also growth inhibited cells that expressed high levels of brain GP but not cells expressing low levels of brain GP [19]. CP-91149 binds at a site located at the subunit interface in the region of the central cavity of the dimeric structure and stabilizes the inactive form of GP [20-23], These observations raised the possibility that glycogen metabolism, and in particular brain GP, may be a potential target for anticancer therapy. Therefore, to understand the regulation and role of glycogen metabolism in prostate cancer in response to androgen we measured intracellular glycogen stores, the activities of GS and GP and G-6-P in prostate cancer cell lines. Our results indicate that glycogen accumulation and reduction in cell growth are associated with the androgen response of prostate cancer cells and can be further enhanced by GP inhibition with the GP inhibitor CP-91149. Thus androgen-dependent growth arrest and cell death can be further enhanced by GP inhibition.

## Methods

### Cell lines and cell culture

The construction and characterization of PC3 cells reconstituted with the androgen receptor (AR) has been reported [24]. For these experiments, PC3-AR cells were stably transfected with vector pZ16E67 BN containing the human papilloma virus E7 protein cDNA (PC3-AR-E72 and E73) or vector pZipNeoSV(X)1 alone (PC3-AR-V1 and V2)[25]. LNCaP cells were obtained from ATCC and experiments were performed with cells around passage 23. All cells were grown in RPMI 1640 lacking phenol red supplemented with charcoal-stripped 5% fetal bovine serum and containing penicillin (100 units/ml), and streptomycin (100 μg/ml). Stably transfected PC3-AR-E7 or V cells were maintained with 100 μg/ml hygromycin and 500 μg/ml G418. Cultures of LNCaP cells were supplemented with 4 nM of the stable testosterone derivative R1881.

Androgen response was induced by either adding 4 nM R1881 (PC3-AR-E7 or V) or by omitting androgen from the culture medium (LNCaP). PC3-AR-E7 or V cells were plated in a density of 5 × 10^5 ^cells per 100 mm dish and LNCaP in a density of 3 × 10^5 ^cells per 100 mm dish. Cells were kept under 95% air and 5% CO_2 _at 37°C in a humidified incubator.

### Glycogen synthase (GS), phosphorylase (GP) activity assays, glycogen and G-6-P determination

GS assays were performed as described with some modifications [26]. For the assay cells from one 100 mm plate were harvested, washed in PBS and lysed in lysis buffer (50 mM Tris/HCl (pH 7.5), 250 mM NaCl, 0.1% NP40, 5 mM EDTA, 50 mM NaF, 1 mM for phenyl-methyl-sulfonyl-fluoride, protease inhibitor cocktail (Boehringer-Mannheim)). Cell lysates were diluted equally with GS dilution buffer. The reaction was started by adding 25 μl assay buffer to 25 μl of the lysate. The assay buffer contained 5 mM UDP-glucose and 1 μCi/ml of [*U*-14*C*]UDP-glucose. 10 mM G-6-P was added to determine the activity of allosterically activated GS. The reaction was performed at 30°C for 15 min and stopped by pipetting the mixture on a p81 Whatman filter. The filters were immersed in 66% EtOH, washed several times in EtOH, once in acetone, air-dried and counted in a liquid scintillation counter (LKB Wallac). GP activity and glycogen content were determined as described with modifications [17,27,28]. The G-6-P concentration was determined in the following way. Nine 100 mm petri dishes were prepared for the isolation of G-6-P and cells collected, sedimented by centrifugation and 0.75 ml 6% perchloric acid added to the cell pellet. Samples were homogenized, iced for 10 min, vortexed and centrifuged at 6000 rpm for 15 min in the Sorvall at 4°C. 0.6 ml of supernatant was neutralized by adding 134 ul of 4 M KOH/0.8 M imidazole. A pH around 7 was confirmed with pH paper. Samples were then centrifuged for 20 min to precipitate the salts. The supernatant was stored at -80°C until assayed in a Hitachi Fluorometer. The reaction was started by adding G-6-P dehydrogenase from *Leuconostroc mesenteroides *and incubated at room temperature for 25 min. Samples were measured before the reaction and immediately afterwards. The excitation wavelength was 365 nm and the emission wavelength 435 nm.

### Flow cytometric analysis

Cells were fixed with 70% ethanol, DNA was stained with propidium iodide. The intensity of fluorescence was measured using a Becton Dickenson flow cytometer at 488 nm for excitation and at 650 nm for emission [29]. The cell cycle profile was analyzed using Modifit's Sync Wizard (Verity Software Inc.).

## Results

### Growth, cell cycle arrest and glycogen content in PC3-AR-E7 and PC3-AR-V cells treated with R1881

**Figure 1:**
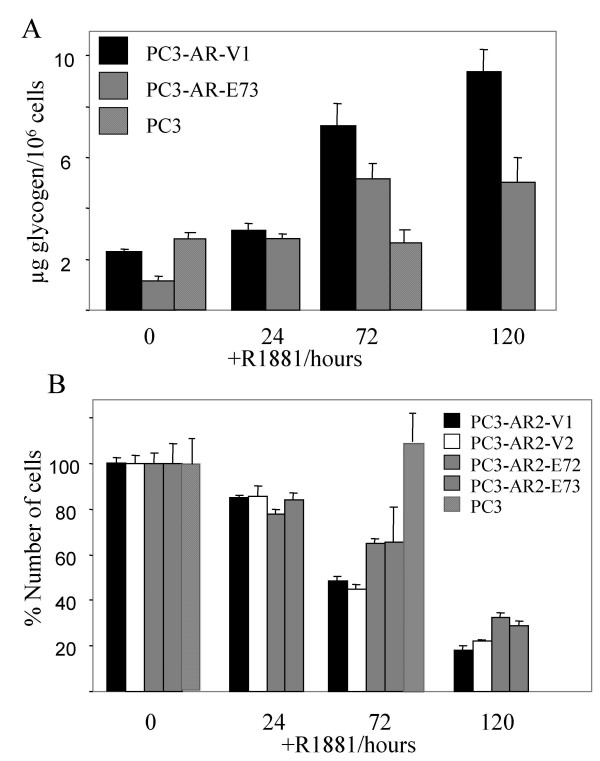
*The effect of R1881 on viable cell number, cell cycle and glycogen content of PC3-AR-V and PC3-AR-E7 cells*. Two independently isolated control (PC3-AR-V1 and PC3-AR-V2) or HPV-E7 expressing clones (PC3-AR-E72 and PC3-AR-E73) were tested. R1881 was added to exponentially growing cells to a final concentration of 4 nM and cells were further incubated for the indicated times. PC3 cells, which lack AR were used as a negative control. Cell samples were removed A) to determine the glycogen content and B) to determine the viable cell count using the trypan blue assay.

The effects of androgen on glycogen metabolism in prostate cancer were investigated using the PC3-AR model [30,31]. PC3 cells do not express the androgen receptor (AR) and are, therefore, androgen-independent. Paradoxically, when PC3 cells are reconstituted with AR (PC3-AR), they become sensitive to the addition of androgen by exiting from the cell cycle and undergoing apoptosis [24]. This phenomenon is until now unexplained. Therefore, to induce an androgen response, the androgen R1881 was added to PC3-AR (PC3-AR-V) cells. Glycogen content normalized to cell count was determined after different incubation times (Fig. 1A). Glycogen content doubled when cells were incubated with R1881 for 24 hours and further increased upon longer incubation times with R1881. This effect was not observed in PC3 cells lacking AR and treated with R1881 for up to 72 hours.

Androgen-independent prostate cancer frequently acquires the loss of a functional retinoblastoma protein (pRb) [32]. Loss of pRb leads to a reduction of androgen-dependent gene expression, which has been interpreted as a possible mechanism for the androgen-independent growth of these cells [33]. Therefore, we tested whether inactivation of pRb reverses the sensitivity of PC3-AR cells to androgen with respect to glycogen accumulation. PC3-AR cells were constructed that expressed the HPV-E7 protein, which binds and inactivates pRb (PC3-AR-E7) [34,35]. PC3-AR cells transfected with the empty vector (PC3-AR-V) were used as a control. As shown in Fig. 1A, PC3-AR-E7 cells show a similar 2 to 5 fold increase in the glycogen content as do the control cells lacking E7 expression. The two cell lines differ in their basal glycogen content, which is about 50% lower in PC3-AR-E7. At 120 hours we observed less glycogen accumulation in the PC3-AR-E7 cells compared to control.

**Figure 2:**
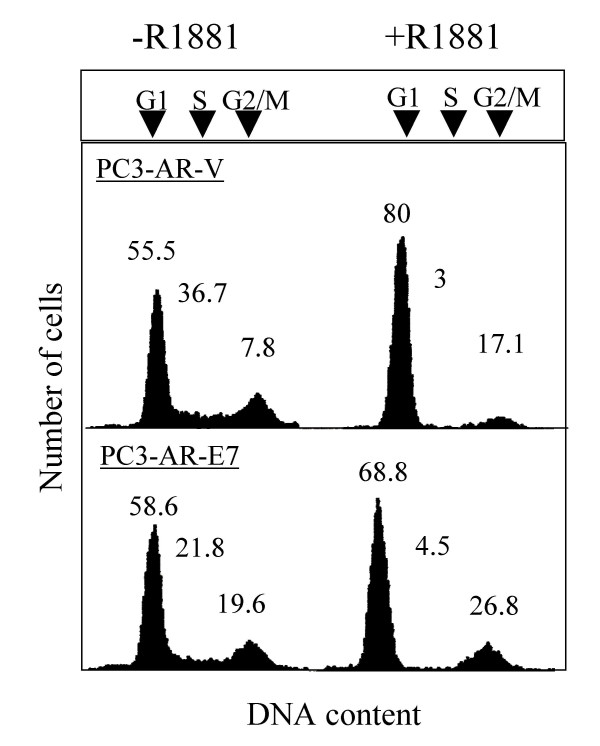
*FACS analysis of R1881-treated PC3-AR-V and PC3-AR-E7 cells*. R1881 was added to exponentially growing cells to a final concentration of 4 nM and cells were further incubated for 24 hours. Cells were then harvested and processed for FACS analysis.

We further compared the number of viable cells using manual cell counts coupled with trypan blue staining when the cell lines were treated with R1881 (Fig. 1B). We tested two E7 and two control transfectants. A gradual reduction in cell number was observed both in PC3-AR-V1/V2 and PC3-AR-E72/E73 cells upon R1881 treatment. However, the PC3-AR-E7 clones showed slightly higher viable cell counts than the control clones following 72 and 120 hours of R1881 treatment. Growth inhibition was not observed for R1881-treated PC3 cells lacking AR and demonstrates that it requires the presence of AR. Cell cycle arrest for both cell lines 24 hours upon R1881 treatment was identified using FACS analysis (Fig. 2). The number of S-phase cells decreased in both R1881-treated PC3-AR cell lines while the number of G1-phase cells increased.

### GS activity in PC3-AR cells treated with R1881

**Figure 3:**
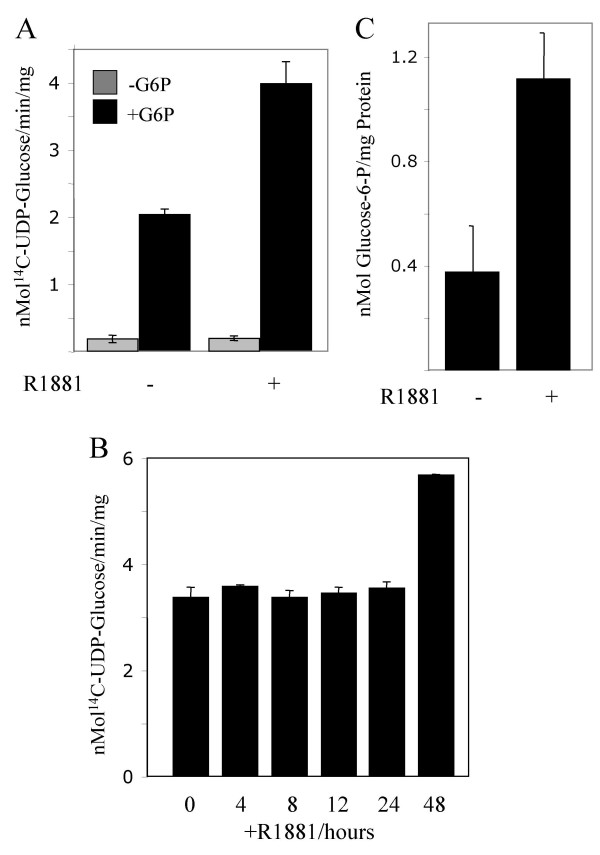
*Determination of glycogen synthase activities in PC3-AR-E7 cells*. R1881 was added to cells to a final concentration of 4 nM. Samples were removed at the indicated times. Activities are in nMol/min/mg protein. A) GS activity was determined from cells treated with R1881 for 72 hours and from untreated cells. GS activities were determined in the absence (-G-6-P) and presence (+G-6-P) of G-6-P. B) GS activities in the presence of G-6-P was determined from cells treated with R1881 for the times indicated. C) The G-6-P content was determined in untreated and 24 hours R1881-treated PC3-AR-E7 cells.

The increase in glycogen content suggests enhanced glycogenesis in androgen-treated PC3-AR cells. The enzyme catalyzing glycogenesis is GS, which exists in two forms, an active unphosphorylated form, and an inactive phosphorylated form, which can be allosterically activated by G-6-P [10]. To determine GS activity, cell extracts were prepared from PC3-AR-E7 cells, which were either untreated or treated with R1881 for 72 hours. GS activity measured in the absence of G-6-P was hardly detectable in untreated and R1881-treated cells suggesting that GS was predominantly present in the inactive phosphorylated state despite hormonal treatment of cells (Fig. 3A). When the GS assay was performed in the presence of G-6-P, which allosterically stimulates GS, an 18 and 35 fold higher GS activity was observed in control and R1881-treated cells respectively. To determine whether the increase in G-6-P activated GS occurs early upon exposure of cells to R1881 PC3-AR-E7 cells were harvested for GS assays in short time intervals (Fig. 3B). G-6-P-activated GS did not show an increase compared to untreated cells (0) until 48 hours after exposure of cells to R1881 indicating that the increase in GS activity is not an immediate response to androgen. However, the observed twofold glycogen increase within 24 hours upon R1881 treatment suggests the possibility that androgen-treatment results in an increase in the intracellular G-6-P content, which allosterically stimulates GS activity. We determined the G-6-P levels in untreated and in 24 hours R1881-treated PC3-AR-E7 cells. We found a 3 fold increase in the G-6-P content normalized to mg protein (Fig. 3C). These results indicate that enhanced glycogenesis in R1881-treated could be the result of GS stimulation by G-6-P.

### GP activity in PC3-AR cells treated with R1881

**Figure 4:**
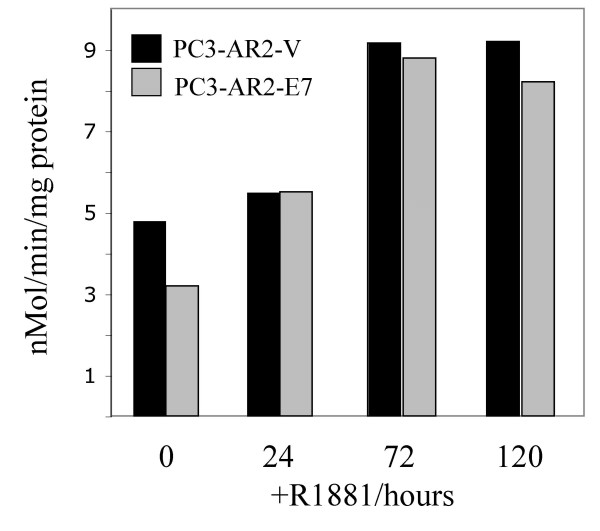
*Glycogen phosphorylase activity in PC3-AR-V/E7 cells*. R1881 was added to cells to a final concentration of 4 nM and GP activities were determined in the absence of AMP, which reflects the activities of the levels of activated GP. The *in vitro *enzyme activity for each sample was followed over a time period of 5 min. The activities are expressed in nMol/min/mg protein.

Glycogen accumulation can also result from inhibition of glycogenolysis, which involves GP. GP exists in two forms, an active phosphorylated (GP-a) and an inactive form (GP-b), which can be regulated by allostery [9]. We examined the GP-a activity in untreated and R1881-treated cells. The GP-a activity normalized to protein in R1881-treated PC3-AR-V and PC3-AR-E7 cells increased slightly within 24 hours and doubled after 72 hours compared to untreated cells demonstrating activation of GP by androgen (Fig. 4). Since despite an increase in GP-activity the glycogen content increased, this suggests steady-state increase in glycogenesis. It is likely that GP-a may be partially inhibited allosterically by G-6-P, which increases in R1881-treated cells.

### Glycogenolysis inhibition by CP-91149 in R1881-treated PC3-AR cells

**Figure 5:**
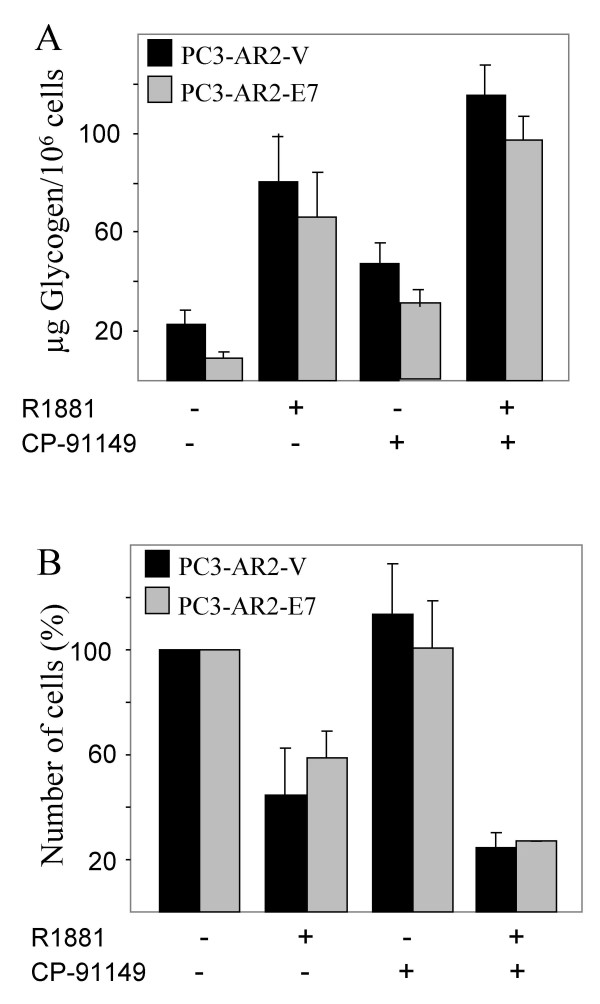
*Determination of viable cell number and glycogen content in PC3-AR-V and PC3-AR-E7 treated with R1881 and/or CP-91149*. Cells were either treated with 4 nM R1881 or 30 μM CP-91149 or both simultaneously. Control cells are those grown in the absence of R1881 and CP-91149 and were harvested after 24 hours. Treated cells were harvested 48 hours later and the total incubation time is 72 hours. A) The glycogen content normalized to the number of cells and B) the number of viable cells was determined.

We showed that stimulation of glycogenesis, which exceeds glycogenolysis appeared to be responsible for the glycogen accumulation that inversely correlated with cell growth upon androgen-treatment in PC3-AR-V and PC3-AR-E7 cells. To test whether the inhibition of glycogenolysis further enhances the cellular response to androgen, GP activity was blocked with the selective GP inhibitor CP-91149. CP-91149 allosterically stabilizes the inactive conformation of human liver GP and induces dephosphorylation of GP-a [21-23]. We previously showed that CP-91149 also inhibits brain GP, which is the GP isoform expressed in all cell lines we have so far analyzed including PC3 cells [19]. PC3-AR-V and PC3-AR-E7 cells were treated with 30 μM CP-91149, which is a sufficient concentration to inhibit GP in several different cell lines including PC3 [[19] and data not shown]. PC3-AR-V and PC3-AR-E7 cells were both growth-arrested in the presence of CP-91149 without androgen-treatment (Fig. 5B). In this particular experiment, untreated cells were harvested 24 hours after the start of the incubation to avoid the effect of high cell density while treated cells were harvested after 72 hours. The actual number of cells from the untreated culture would, therefore, be much higher after 72 hours compared to the CP-91149-treated culture (about double). This demonstrates inhibition of cell growth by CP-91149 treatment alone. The cell number of R1881-treated cultures was about 50% smaller compared to CP-91149-treated cultures. When cells were treated with both R1881 and CP-91149 simultaneously, the cell number was reduced to about 50% of R1881-treatment alone consistent with an additive effect of CP-91149 on R1881-treatment. These results indicate that glycogenolysis in cells treated with R1881 is still enough to support growth and/or survival as the intracellular glycogen content correlated inversely to the number of cells (Fig. 5A). Untreated cells had the lowest glycogen content as normalized to cell count while the glycogen level doubled in CP-91149-treated cells. A 4-7 fold increase in the glycogen content was achieved by R1881-treatment. CP-91149 and R1881-double treatment resulted in 35–40% additional increase in glycogen content. This result further confirmed a tight correlation between glycogen accumulation and cell growth of R1881-treated PC3-AR cells and demonstrated that blockage of glycogenolysis by inhibition of GP-a with CP-91149 enhanced the effect of androgen on both PC3-AR-V and PC3-AR-E7 cells.

### R1881 withdrawal results in glycogen accumulation in LNCaP cells

**Figure 6:**
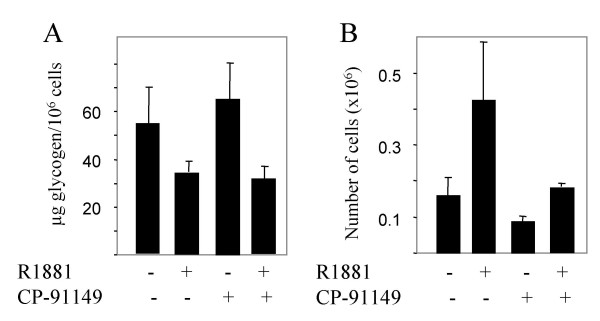
*Determination of viable cell number and glycogen content of LNCaP cells deprived of R1881 in the absence or presence of CP-91149*. Control cells were grown in the presence of R1881. Treated cells were either deprived of R1881 and/or treated with 30 μM CP-91149. The total length of the experiment was seven days. A) The glycogen content was determined and normalized to the number of cells. B) The number of viable cells was determined.

The effects of androgen on the glycogen content was then evaluated in the androgen-dependent prostate cancer cell line LNCaP. The LNCaP cell line expresses a functional AR endogenously and growth arrests in the absence of androgen. LNCaP cells were cultured for seven days in the presence and absence of R1881 with regular medium changes. Cells ceased to grow and the intracellular glycogen content increased compared with cells grown in the presence of R1881 (Fig. 6). LNCaP cells were similarly growth-arrested as PC3-AR cells when glycogenolysis was inhibited with CP-91149 (Fig. 6B). We observed no difference in the glycogen level from control cells grown in the presence of R1881 and absence of CP-91149. The most likely explanation is that control cells were grown to high density, which has been reported to result in decreased GP-a activity in colon cancer cells [36]. When R1881 was removed from the medium and simultaneously CP-91149 was added, the number of cells further reduced and the cell count normalized to the glycogen level increased to a higher extent than either treatment alone. Thus the inverse relationship between intracellular glycogen accumulation and cell growth and the additive effect of GP inhibition in androgen-deprived cells were reproduced in the androgen-dependent prostate cancer LNCaP cell line.

## Discussion

In this paper we describe the effect of androgen on glycogen metabolism in different prostate cancers. We used PC3-AR-V and PC3-AR-E7 cells, which ectopically expresses AR and in case of PC3-AR-E7 the HPV-E7 protein. Additionally, LNCaP, which maintains a functional AR, was tested for glycogen content and its correlation to the number of cells. The PC3-AR-V and PC3-AR-E7 cell lines demonstrated a response to androgen leading to G1 arrest with a corresponding increase in the glycogen content (2 to 5 fold). PC3 cells lacking AR did not increase glycogen content in response to androgen. PC3 (AR-) cells demonstrate that intracellular glycogen content corresponds with the growth and/or survival of cells harbouring a functional AR. Intracellular stores of glycogen correlated inversely with the cell number: when cell numbers are low the glycogen content is high. This inverse relationship suggests that glycogenesis participates in growth arrest. However, glycogenesis is most likely not sufficient to induce ATP-dependent apoptosis, as the inhibition of glycogenolysis with the GP inhibitor CP-91149 does not induce cell death in these prostate cell lines. Glycogen content normalized to the number of cells is about 50% higher in PC3-AR cells treated with R1881 than treated with CP-91149. The additional increase in glycogen content of R1881-treated PC3-AR-V/E7 cells upon CP-91149 treatment further results in a reduction of cell number by growth inhibition and reduction in cell viability, which suggests that a certain intracellular glycogen content has to be reached to affect cell viability. Alternatively, certain effects of hormone treatment on cell survival may be enhanced by inhibition of glycogenolysis using CP-91149. Similarly, LNCaP cells responded with glycogen accumulation and growth arrest upon androgen removal, which was further enhanced by the GP inhibitor CP-91149.

One explanation of reduced cell viability in conjunction with glycogen content could be the increase in G-6-P in response to androgen. G-6-P is a key metabolite allosterically activating GS [12]. The G-6-P content was about 3 fold higher 24 hours after androgen addition in PC3-AR cells compared to untreated cells. The increase in the G-6-P level was most probably a consequence of the decrease in activity of some key glycolytic enzymes as well as G-6-P dehydrogenase, which have been reported to be under androgen control [3]. We can, however, not exclude the presence of another allosteric GS activator nor partial activation of GS, which would sensitize GS to allosteric activation. The increase in G-6-P could also explain why an increase in activated GP (GP-a) in PC3-AR cells upon androgen treatment did not prevent the increase in glycogen content, since G-6-P is a GP-a inhibitor [37,38]. However, the fact that CP-91149 increased the glycogen content twofold more in androgen-treated PC3-AR cells shows that there was still GP-a activity. CP-91149 is known to cause GP-a dephosphorylation and partial GS-activation [39].

## Conclusion

Glycogenesis is part of the androgen response in prostate cancer and further inhibition of glycogen phosphorylase by a specific inhibitor reduces the cell number suggesting that glycogenolysis contributes to cell survival. Thus inhibition of glycogenolysis in combination with hormone therapy may be a more effective treatment for advanced prostate cancer than hormone therapy alone.

## List of abbreviations used

GP, Glycogen Phosphorylase;

GS, Glycogen Synthase;

G-6-P, Glucose-6-Phosphate;

AR, Androgen Receptor.

HPV-E7, Human Papilloma Virus E7

## Competing interests

The author(s) declare that they have no competing interests.

## Authors' contributions

J.S. is the corresponding author. His main contributions are the idea to carry out this research, experimental design and carrying out most of the experiments.

K.N. has carried out some experiments and contributed conceptually.

P.G. has funded some experiments, contributed materials used for the experiments and contributed with his expertise in prostate cancer research.

F.G. has contributed with some funding and was a collaborator regarding some experiments.

E.M.B. has provided funding, lab space and was a consultant.

## Pre-publication history

The pre-publication history for this paper can be accessed here:


